# Psychometric Properties of the Children With Cerebral Palsy (7–18 Years Old) Self‐Care Skills Scale‐Parent Form: A Turkish Validity and Reliability Study

**DOI:** 10.1002/nop2.70477

**Published:** 2026-03-19

**Authors:** Betul Yavuz, Bircan Kahraman Berberoğlu, Hüsniye Çalişir

**Affiliations:** ^1^ Faculty of Health Sciences, Department of Pediatric Nursing Kütahya Health Sciences University Kütahya Türkiye; ^2^ Nursing Faculty, Department of Pediatric Nursing Aydın Adnan Menderes University Aydın Türkiye

**Keywords:** cerebral palsy, child, self‐care

## Abstract

**Aim:**

This study aimed to develop the children with cerebral palsy (7–18 years old) self‐care skills scale‐parent form and assess its validity and reliability.

**Design:**

The sample of this methodological study consisted of 317 parents of children with cerebral palsy (CP) aged 7–18 years who were trained in special education and rehabilitation centres.

**Methods:**

The data were collected using the Child–Parent Information Form and the Children with Cerebral Palsy (7–18 Years Old) Self‐Care Skills Scale‐Parent Form (CCPSCSS‐PF). The data were analysed using descriptive statistics, Kruskal‐Wallis, Cronbach's alpha, test–retest, the Kaiser‐Meyer‐Olkin (KMO) test, Bartlett's test, exploratory factor analysis (EFA), and confirmatory factor analysis (CFA).

**Results:**

The Cronbach's alpha coefficients for the overall scale and its two subscales were 0.971, 0.984, and 0.913 and the test–retest reliability coefficient of the overall scale was 0.976. The KMO sample fit coefficient of the scale was 0.948, and Bartlett's test of sphericity *χ* ^2^ value was 8472.344 (*p* < 0.001). According to the EFA results, the scale items were grouped under two factors. The factor loadings of the CCPSCSS‐PF ranged from 0.767 to 0.951 for Factor 1 (self‐care) and 0.811 to 0.950 for Factor 2 (ability to express self‐care). The scale accounted for 83.4% of the total variance. The fit indices calculated in CFA were 1.000 for GFI, AGFI, CFI, TLI, and IFI; 0.097 for RMSEA; and 2.489 for CHISQ/Df.

**Conclusion:**

The CCPSCSS‐PF is a valid and reliable assessment tool that can be used to evaluate the self‐care skills of children with CP aged 7–18 years.

**Implications for Practice:**

The CCPSCSS‐PF can be used in preventive and rehabilitative studies by professionals caring for children with CP.

## 1 Introduction

Cerebral palsy (CP) is a group of permanent disorders resulting from damage to the developing foetus or infant brain, causing permanent but non‐progressive activity limitations and leading to problems in mobility and postural development (Himmelmann and Panteliadis 2018; Ferriero and Arn 2020; Fritz and Sewell‐Roberts 2020). Motor problems, the fundamental problem of children with CP, are often accompanied by secondary disorders (mental retardation, epileptic seizures, dental, hearing, visual, respiratory, gastrointestinal, urinary, musculoskeletal, oral motor, sleep, and behavioural problems, and pain) (Himmelmann and Panteliadis 2018; Sadowska et al. 2020).

The ability to perform self‐care skills is correlated with the development of motor skills. Children who develop normally usually begin to perform self‐care skills independently from the age of 7 (Chen 2019). The ability of children with CP to perform self‐care skills is affected by the child's age, the level of gross motor function, bimanual arm function, visual function, mental capacity (de Leeuw et al. 2021), hearing, speech, perceptual and behavioural disorders, and convulsion conditions. Therefore, it is important that each child is evaluated in accordance with their physical and cognitive capacity (Kunz Stansbury and Wilson 2015; Chen 2019). Nurses are among the healthcare professionals who establish one‐to‐one communication with children with CP and their families at most in clinics. Nurses can evaluate children's ability to perform self‐care skills and play a role in the correction of misbehaviours and the acquisition of positive skills (Cook et al. 2022; Kahraman Berberoğlu and Çalışır 2020; Zahavi and Martiny 2019).

There are assessment tools used by specialists (occupational therapists, physiotherapists, physicians, etc.) to assess the self‐care of children with CP. These tools include the Gross Motor Function Classification System (GMFCS), Barthel Index, Paediatric Functional Independence Measure, Paediatric Evaluation of Disability Inventory‐Computer Adaptive Test (PEDI‐CAT), Functional Mobility Scale (FMS), Manual Ability Classification System in Children with Cerebral Palsy (MACS), and Gross Motor Function Measure Score Sheet (GMFM 88 and GMFM 66) (Bobath Therapists Association 2024). However, these assessment tools are not specific to the age and developmental periods of children, are used by professionals, and are not suitable for the use of parents. A questionnaire developed by Yavuz and Çimen to assess the self‐care skills of children with CP is available in the literature (Yavuz 2006). However, this form can be applied to children with CP aged 7–18 years, but its validity and reliability have not been established.

Although various assessment tools are available and commonly used by clinicians to evaluate the psychomotor and daily living skills of children with cerebral palsy (CP), there is a clear need for a tool that enables parents, nurses, and other healthcare professionals to assess the self‐care abilities of children with CP. In this context, the *Self‐Care Assessment Parent Form for Children with Cerebral Palsy Aged 7–18* was developed to allow parents to actively observe and evaluate their children's self‐care skills. This involvement supports parents in playing an informed and effective role in the care process led by nurses, especially within the framework of family‐centered care. Therefore, there is a need for a valid and reliable assessment tool that can be used by parents to assess the self‐care of children and adolescents with CP in the age group of 7–18 years. This study was conducted to test the validity and reliability of the scale we developed to assess the self‐care of children with CP aged 7–18 years based on parent reports.

## 2 Methods

### 2.1 Aim and Design

This methodological study was conducted to develop the children with CP (7–18 years old) self‐care skills scale‐parent form and to test its validity and reliability. The findings of the study were presented following the guidelines outlined in the STROBE (see Supporting Information).

Research questions:

Q1. Is the Children with Cerebral Palsy (7–18 years old) Self‐Care Skills Scale‐Parent Form a valid assessment tool?

Q2. Is the Children with Cerebral Palsy (7–18 years old) Self‐Care Skills Scale‐Parent Form a reliable assessment tool?

### 2.2 Setting and Participants

The study was conducted with the parents of children with CP aged 7–18 years who received physical therapy at ten special education and rehabilitation centers in two western provinces of Türkiye between June 22 and December 22, 2023. The sample size in validity and reliability studies is recommended to be 5–10 times the number of items on the scale (Esin 2018). The present study's sample size was calculated by multiplying the total number of items by 10 times (30 items × 10 = 300) after content validity. Non‐probability random sampling method was used for sample selection. The study was completed with 317 parents. The power value calculated by correlation analysis in the G*Power software was 99.979% for *n* = 317 based on a medium effect size (*r* = 0.30) and a Type I error of *α* = 0.05 (Cohen 1988).

The inclusion criteria were determined as follows: (1) being aged 7–18 years for children; (2) cohabiting with their parents; (3) at least literate for parents; and (4) being able to speak and understand Turkish. The exclusion criteria were as follows: (1) having hearing, visual, or speech disability for the parents; and (2) having cognitive disability.

### 2.3 Data Collection Tools

The data were collected using a ‘Child‐Parent Information Form’ and ‘the Children with Cerebral Palsy (7–18 years old) Self‐Care Skills Scale‐Parent Form (CCPSCSS‐PF)’.

#### 2.3.1 Child–Parent Information Form

This form included socio‐demographic characteristics (age, gender, educational background, socio‐economic level, etc.) of the children with CP and their parents included in the study, as well as information about their disease (Yavuz and Çimen 2007).

#### 2.3.2 Children With Cerebral Palsy (7–18 Years Old) Self‐Care Skills Scale‐Parent Form (CCPSCSS‐PF)

Based on the literature, the researchers developed the draft version of the CCPSCSS‐PF to assess self‐care performance in children with CP aged 7–18 years (Yavuz and Çimen 2007). Initially, the scale contained 42 items, which were reviewed by experts from various paediatric fields. Following expert feedback, 12 items were removed and some were revised, reducing the scale to 30 items. Exploratory factor analysis led to the exclusion of an additional 11 items, resulting in a final scale of 19 items grouped under two factors: Factor 1 (self‐care) with 16 items and Factor 2 (ability to express self‐care) with 3 items. The self‐care items cover skills such as feeding, toileting, dressing, and personal hygiene, while the expression items assess the child's ability to communicate needs like hunger, thirst, and toileting. The scale uses a 5‐point Likert format ranging from 1 (‘Never’/completely dependent) to 5 (‘Always’/independent), with four items (items 3, 10, 27, and 28) reverse‐scored. Total scores range from 19 to 95, where higher scores indicate better self‐care abilities.

### 2.4 Pilot Study

A pilot study with the final Turkish version of the scale was conducted on 10 parents, and the clarity of the expressions was evaluated. These data were not included in the data analysis.

### 2.5 Data Collection

Data were collected by holding face‐to‐face interviews with the participants. The researchers did not interrupt the children's practices in the rehabilitation centre during data collection. For test–retest reliability analysis, the scale was completed for the second time by holding face‐to‐face interviews with 76 volunteer parents again 2 weeks after the initial data collection.

### 2.6 Data Analysis

The data were analysed using IBM SPSS 27 and R software. Descriptive statistics were reported by using frequency, percentage, mean and standard deviation values. The normal distribution of data was evaluated with the Shapiro–Wilk test. Content validity index (CVI) and exploratory and confirmatory factor analyses were used for validity. Exploratory factor analysis (EFA) was done using a polychoric correlation matrix to identify the subscales of the scale, and the number of subscales was selected using Horn's parallel method. The Varimax was used as the rotation method and principal component analysis as the estimation method (Holgado‐Tello et al. 2010). Confirmatory factor analysis (CFA) was performed using the diagonal weighted least squares (DWLS) method. Reliability analyses included Cronbach's alpha, corrected item‐total correlations, and test–retest reliability. For mean comparisons of variables with more than three groups, the Welch test was run when the groups were normally distributed, and pairwise comparisons between groups that were found to be significant were analysed with the Games‐Howell test when the variances were not homogeneous. These analyses were done using the ggstatsplot (v0.9.0) software (Patil 2021). While EFA was done with approximately 50% of the data, CFA was done with the other 50%. The significance level was accepted as *p* < 0.05.

### 2.7 Ethical Considerations

This study was conducted in accordance with the Helsinki Declaration and was approved by the Nursing Faculty Non‐Interventional Ethics Committee (Date: 22.05.2023, Number: 2023/352). Verbal consent of the participants was obtained. Written permission was obtained from the special education and rehabilitation centres where the research was conducted. The children's practices were not interrupted in the rehabilitation centre during data collection.

## 3 Results

The mean age of the children was 12.26 ± 3.63 years, with 45.7% being girls and 54.3% boys. The mean ages of mothers and fathers were 39.68 ± 6.69 and 43.22 ± 7.28 years, respectively, and approximately one‐third had completed only primary school education. More than half of the families (57.4%) reported that their income was equal to their expenses. A majority of the children (86.4%) had attended the rehabilitation centre for 3 years or longer, with 52.1% attending twice per week. In 68.1% of cases, cerebral palsy was accompanied by comorbid disorders. The mean total score of the CCPSCSS‐PF was 55.00 ± 25.18, with subscale means of 45.17 ± 23.61 for Factor 1 and 9.83 ± 4.66 for Factor 2.

### 3.1 Validity Analysis

#### 3.1.1 Content Validity

To establish the content validity of the raw scale, which initially consisted of 42 items, the opinions of 10 experts from different disciplines in the field of paediatrics (paediatric diseases nurses, child development specialists, paediatric neurologists, physiotherapists, and occupational therapists) were sought to evaluate the fitness and content integrity. The experts were asked to evaluate each item on a scale by assigning a score between 1 and 4 points for fitness and comprehensibility and to report their opinions and suggestions in writing. Based on the suggestions from the experts, 12 items were removed from the scale. Some scale items were corrected, and a draft scale consisting of 30 items was prepared. Then, opinions and suggestions were solicited from a Turkish language expert for the grammatical and expressive integrity of the scale items in Turkish. Necessary corrections were made to some scale items.

Expert opinions were evaluated with the Content Validity Index (CVI). In line with expert opinions, content validity index (I‐CVI, S‐CVI) for items and scale tests was evaluated using the Davis Method. The I‐CVI of the scale items evaluated using the Davis Method ranged from 0.80 to 1.00, and the S‐CVI was found to be 0.91.

#### 3.1.2 Construct Validity

In the study, it was found that the Kaiser‐Meyer‐Olkin (KMO) coefficient of sampling adequacy was 0.948, and the Bartlett's Test of Sphericity *χ* ^2^ value was 8472.344 (*p* < 0.001). These results showed that the data were fit and adequate for factor analysis (Table 1).

**TABLE 1:** KMO measure of sampling adequacy and Bartlett's test values.

KMO measure of sampling adequacy		0.948
Bartlett's test of sphericity	Approx. chi‐square	8472.344
	Df	171
	*p*	**< 0.001**

*Note:* Bold font was used specifically to highlight the statistically significant *p*‐values.

Abbreviation: Df, degrees of freedom.

According to the results of the EFA, 11 items (i4, i5, i9, i17, i18, i19, i20, i23, i28, i29, and i30) with factor loadings less than 0.40 were omitted from the scale (Costello and Osborne 2005). The remaining 19 items of the scale were grouped under two factors (Factor 1, Factor 2). Considering the scale items, the first subscale was called ‘self‐care’ and the second subscale was called ‘the ability to express self‐care’. Factor 1 (self‐care) accounted for the largest portion of the total variance (66.0%), and Factor 2 (ability to express self‐care) accounted for 17.5%. These two factors accounted for 83.4% of the variance. The factor loadings of the scale ranged between 0.767 and 0.951 for Factor 1 and 0.811 and 0.950 for Factor 2. On the other hand, the communality values of the two factors defined in relation to the scale items ranged between 0.713 and 0.908 (Table 2).

**TABLE 2:** Factor loadings, communalities, and variance explanation ratio of the CCPSCSS‐PF.

Items	Item number	F1	F2	Communality
1. My child can put on his/her bottom garments (pants, shorts, pyjamas, etc.) by himself/herself	i25	0.951		0.908
2. My child can put on his/her top garments (cardigans, shirts, t‐shirts, etc.) by himself/herself	i24	0.940		0.889
3. My child can clean herself/himself with toilet paper after using the toilet	i14	0.931		0.877
4. My child can go to the toilet by himself/herself	i11	0.922		0.891
5. My child can put on his/her shoes by himself/herself	i26	0.913		0.835
6. My child can take off and put on his/her clothes in the toilet by himself/herself	i13	0.907		0.863
7. My child can comb his/her hair by himself/herself	i22	0.898		0.813
8. My child can apply toothpaste to the toothbrush by himself/herself	i1	0.886		0.856
9. My child can wash his/her face by himself/herself	i16	0.882		0.880
10. My child can brush his/her teeth by himself/herself	i2	0.877		0.846
11. My child can take a bath by himself/herself	i21	0.864		0.786
12. My child can pour water into a glass by himself/herself	i8	0.862		0.846
13. My child can put on his/her shoes with someone's help	i27	0.844		0.715
14. My child can eat with someone else's help	i3	0.833		0.770
15. My child can wash his/her hands by himself/herself	i15	0.832		0.846
16. My child can drink water with someone else's help	i10	0.767		0.727
17. My child can verbally/nonverbally express or show that he/she is hungry	i6		0.950	0.907
18. My child can verbally/nonverbally express or show that he/she is thirsty	i7		0.944	0.894
19. My child can verbally/nonverbally express or show that he/she needs to go to the toilet	i12		0.811	0.713
	Eigenvalue	12.536	3.325	
	PEV	0.660	0.175	0.834

Abbreviation: PEV, percantage of explained variance.

When the fit index values of the CFA findings of the scale were analysed, it was found that the CHISQ was 350.972, the Df was 141, and the CHISQ/Df ratio was 2.489, and all values of the GFI, AGFI, CFI, TLI, and IFI was 1.000, and the RMSEA value was 0.097 (Table 3).

**TABLE 3:** The fit indices of CFA findings of the CCPSCSS‐PF.

*χ* ^2^	Df	GFI	AGFI	CFI	TLI	IFI	RMSEA
350.972	141	1.000	1.000	1.000	1.000	1.000	0.097

Abbreviations: *χ* ^2^, Chi‐square; AGFI, adjusted goodness of fit index; CFI, comparative fit index; Df, degrees of freedom; GFI, goodness of fit index; IFI, incremental fit index; RMSEA, root mean square error of approximation; TLI, Turker‐Lewis index.

According to the CFA analysis, the scale items were grouped under two factors. The standardised factor loadings of the subscales of CCPSCSS‐PF ranged from 0.801 and 0.968 for Factor 1 (*p* < 0.001) and from 0.357 to 1.834 for Factor 2 (*p* < 0.001) (Table 4).

**TABLE 4:** Factor loadings in the CFA results of the CCPSCSS‐PF.

Factor	Item	Factor loading	Standardised factor loading	*z*	*p*
F1 Self‐care	i25	1	0.968		
	i24	0.991	0.960	92.388	**< 0.001**
	i14	0.953	0.923	88.117	**< 0.001**
	i26	0.971	0.940	86.824	**< 0.001**
	i11	0.982	0.951	100.241	**< 0.001**
	i22	0.894	0.866	70.771	**< 0.001**
	i13	0.997	0.966	98.498	**< 0.001**
	i1	0.939	0.909	80.531	**< 0.001**
	i2	0.927	0.898	75.597	**< 0.001**
	i16	0.970	0.940	89.569	**< 0.001**
	i27	0.854	0.827	58.21	**< 0.001**
	i8	0.946	0.916	84.83	**< 0.001**
	i3	0.868	0.841	63.294	**< 0.001**
	i15	0.950	0.920	79.474	**< 0.001**
	i10	0.843	0.816	55.146	**< 0.001**
	i21	0.827	0.801	51.642	**< 0.001**
F2 Ability to express self‐care	i7	1	0.378		
	i6	0.946	0.357	9.126	**< 0.001**
	i12	4.856	1.834	5.478	**< 0.001**

*Note:* Bold font was used specifically to highlight the statistically significant *p*‐values.

**FIGURE 1:**
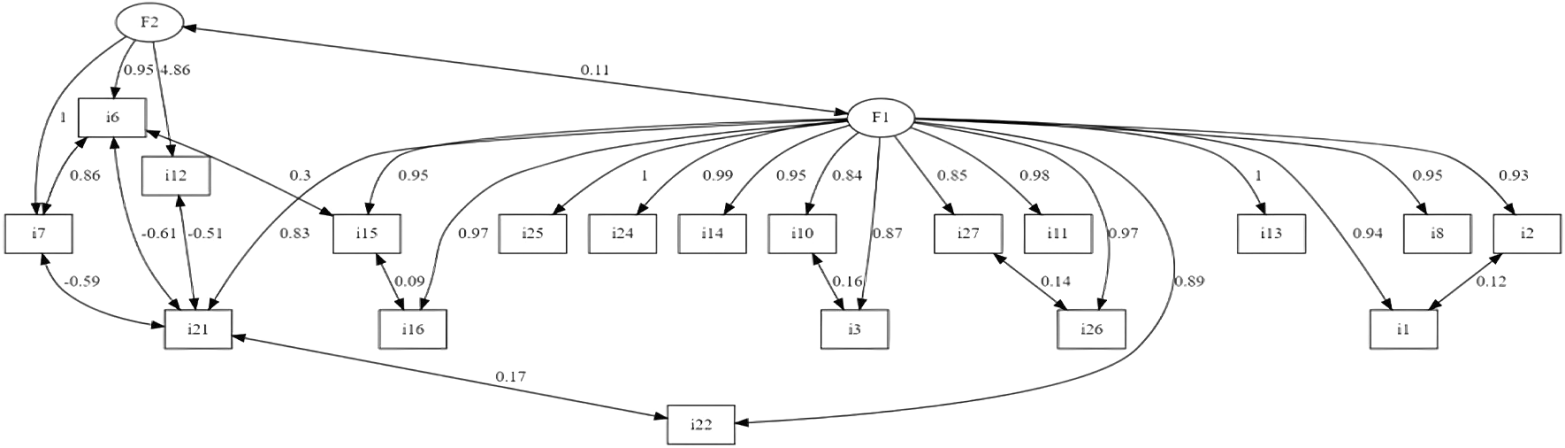
Diagram of confirmatory factor analysis.

The CFA graph shows the factor loadings and inter‐factor covariance values for the scale items (Figure 1).

### 3.2 Reliability Analysis

#### 3.2.1 Internal Consistency

The internal consistency Cronbach's Alpha for the overall CCPSCSS‐PF was 0.971. The subscale reliability coefficients of the scale were α = 0.984 (Omega 0.987) for Factor 1 and α = 0.913 (Omega 0.928) for Factor 2. These results showed that the internal consistency of the scale was high. The corrected item‐total correlation values of the subscales of the scale ranged between 0.808–0.943 for Factor 1 and 0.843–0.966 for Factor 2 (*p* < 0.001). No item was removed from the scale during the reliability analysis (Table 5).

**TABLE 5:** Reliability analysis results of the CCPSCSS‐PF.

Factor	Item	Mean	SD	Corrected‐item total correlation	Alpha if item deleted	Alpha	Omega
F1 Self‐care	i25	2.570	1.679	0.943	0.982	0.984	0.987
	i24	2.582	1.705	0.936	0.983		
	i14	2.589	1.731	0.932	0.983		
	i11	2.684	1.752	0.943	0.982		
	i26	2.703	1.739	0.903	0.983		
	i13	2.633	1.675	0.927	0.983		
	i22	2.633	1.691	0.896	0.983		
	i1	2.854	1.677	0.920	0.983		
	i16	2.842	1.721	0.928	0.983		
	i2	2.880	1.739	0.914	0.983		
	i21	2.449	1.691	0.808	0.984		
	i8	2.861	1.750	0.909	0.983		
	i27	2.734	1.672	0.821	0.984		
	i3	3.057	1.679	0.874	0.983		
	i15	2.905	1.737	0.894	0.983		
	i10	3.146	1.696	0.829	0.984		
F2 Ability to express self‐care	i6	3.196	1.757	0.966	0.794	0.913	0.928
	i7	3.184	1.762	0.956	0.814		
	i12	3.101	1.656	0.843	0.988		

Abbreviation: SD, standard deviation.

#### 3.2.2 Invariance Test/Retest

To establish the time‐dependent consistency and invariance of the scale, 76 parents who volunteered for test–retest reliability analysis filled out the scale once more two weeks later. According to the test–retest reliability analysis, a positive, significant, and strong correlation was found between the total scores of the scale obtained at two different times (*r* = 0.976, *p* < 0.001). This result supported that the scale is a reliable assessment tool for the overall construct.

There were statistically significant differences between the total mean score of the CCPSCSS‐PF, the mean scores of Factor 1 (self‐care) and Factor 2 (ability to express self‐care), and the GMFCS levels.

**FIGURE 2:**
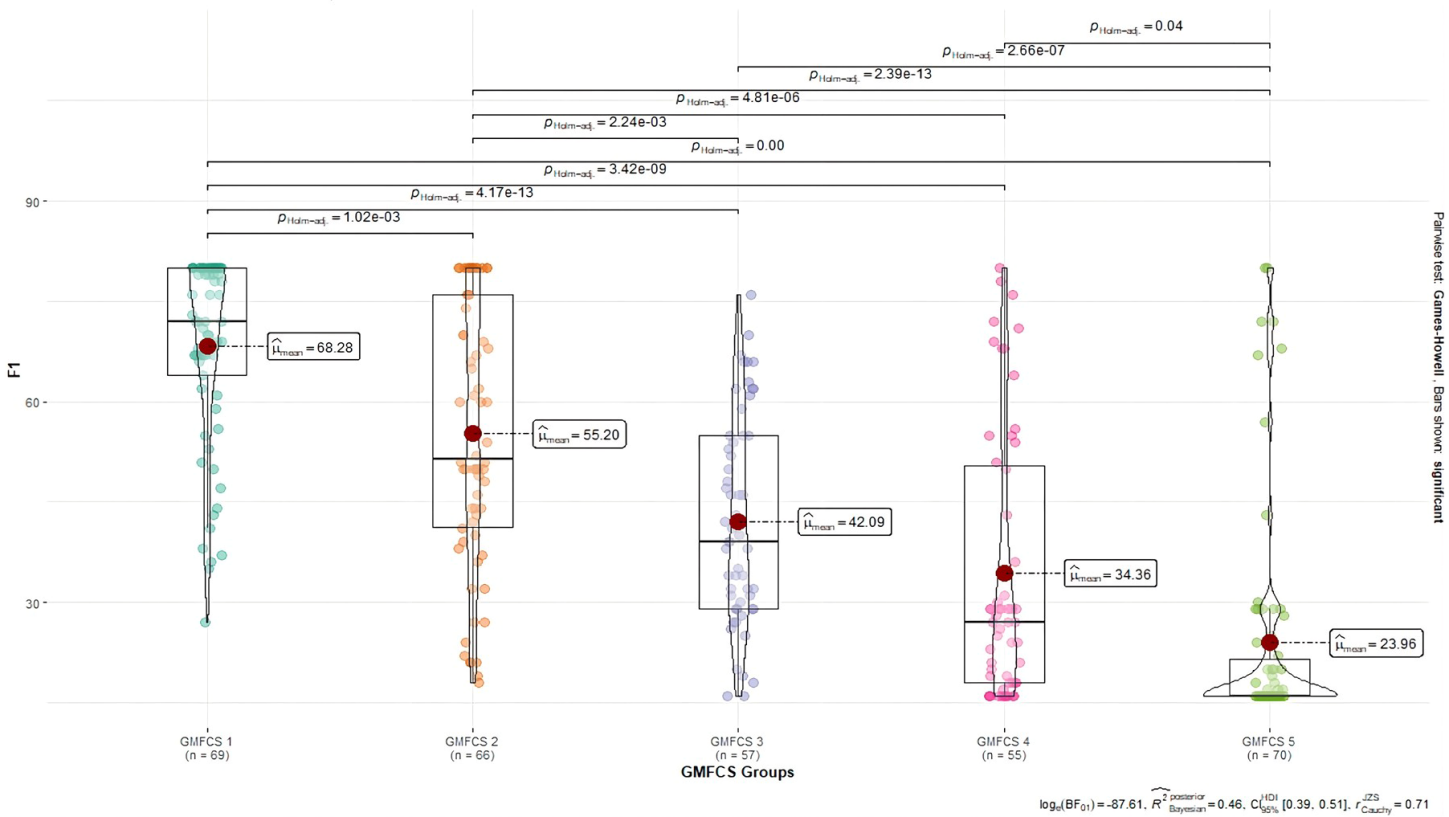
Comparisons of Factor 1 scores according to GMFCS levels.

For Factor 1, the self‐care mean score of children with CP was the highest at GMFCS Level 1 and the lowest at GMFCS Level 5. As we progressed from GMFCS 1 to GMFCS 5, the self‐care mean scores of the children decreased significantly for Factor 1 (Figure 2).

**FIGURE 3:**
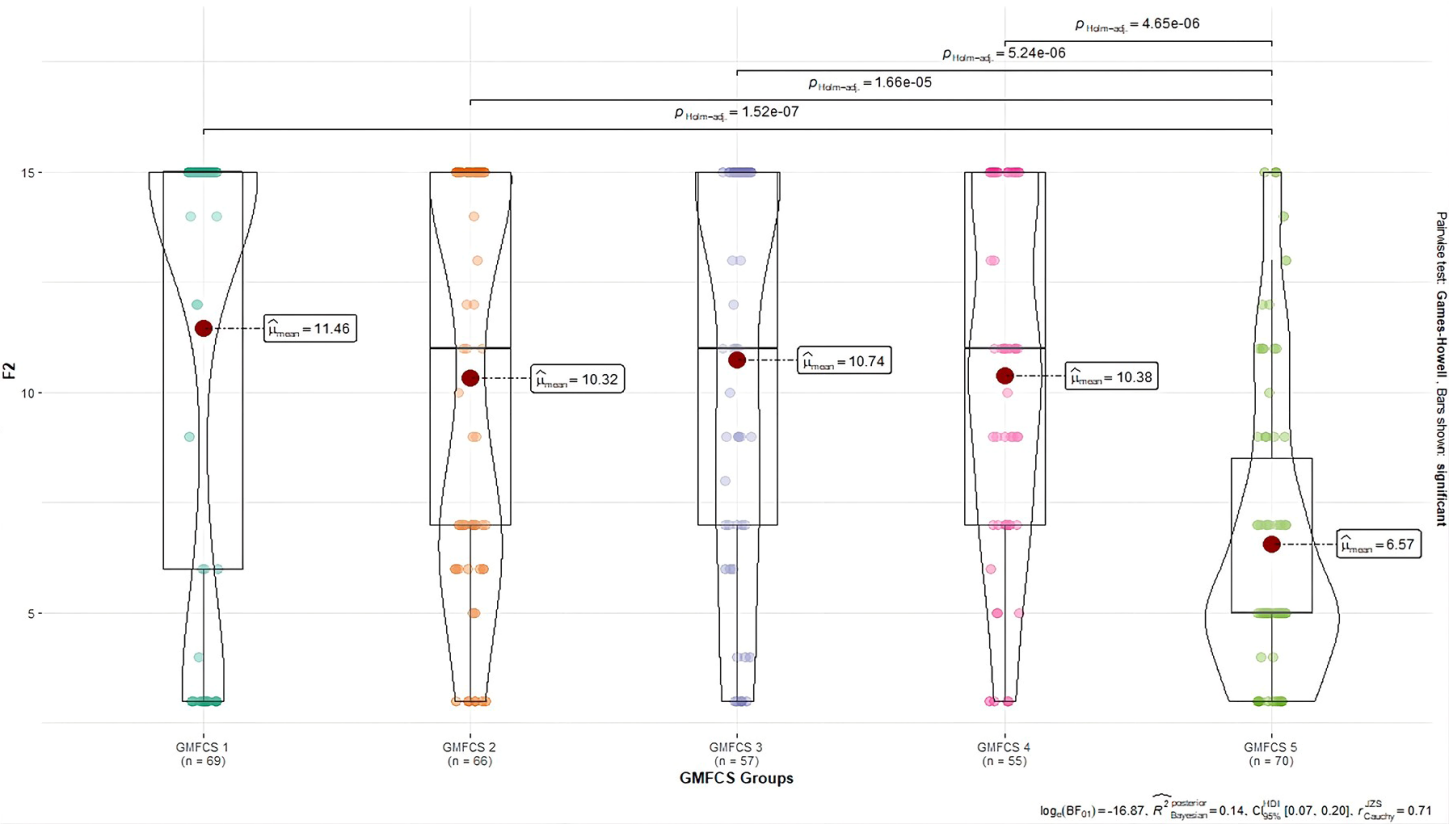
Comparisons of Factor 2 scores according to GMFCS levels.

For Factor 2, the self‐care mean scores of children with CP were significantly lower at the GMFCS Level 5 compared to the other levels and this difference was statistically significant. Moreover, the self‐care mean scores of children for Factor 2 differed among other GMFCS levels, but the most significant drop was found at GMFCS Level 5 (Figure 3).

**FIGURE 4:**
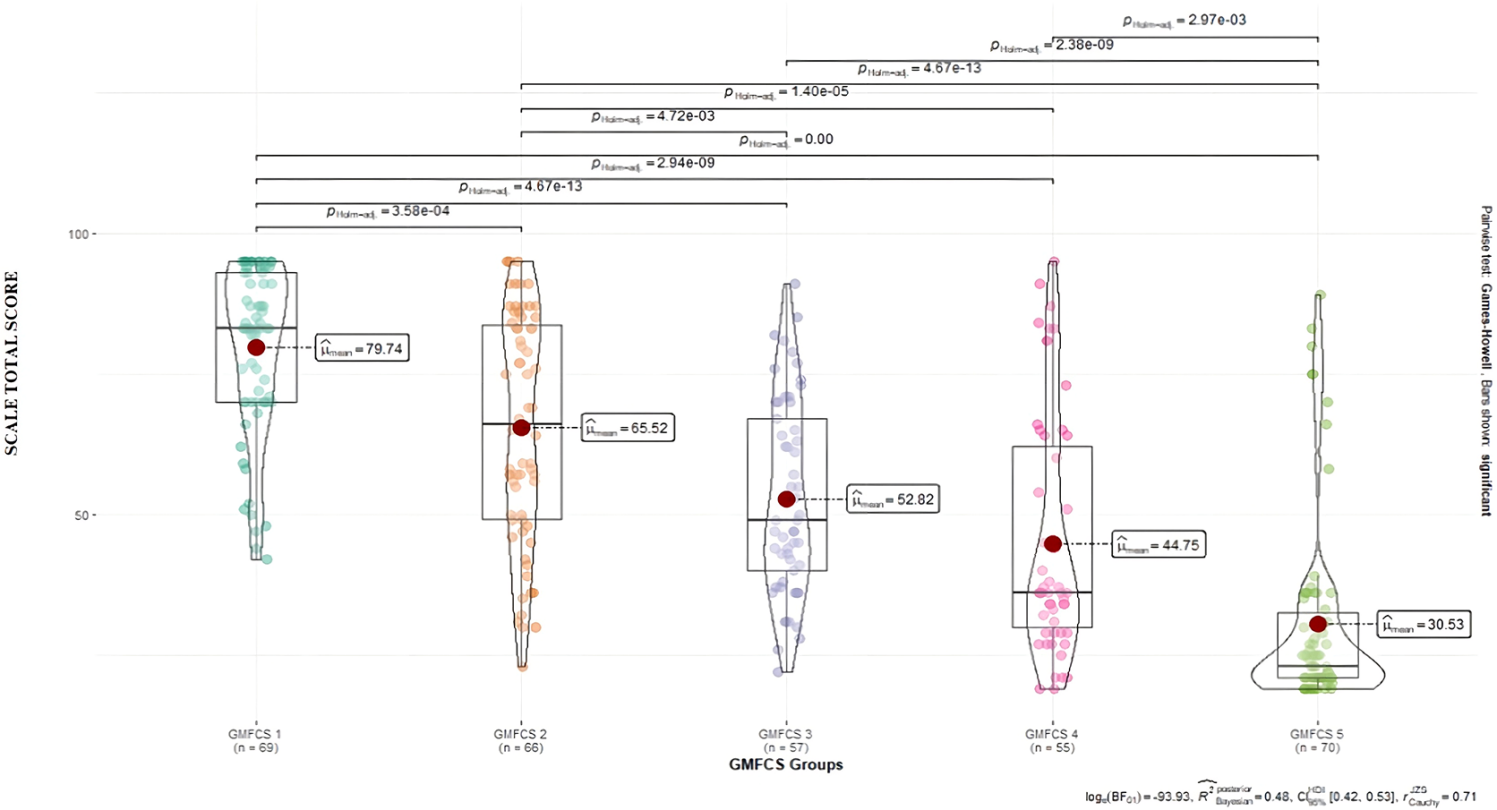
Comparisons of scale total scores according to GMFCS levels.

For the overall CCPSCSS‐PF, the CCPSCSS‐PF total mean score of children with CP was the highest at the GMFCS Level 1 and lowest at the GMFCS Level 5. As the GMFCS level increased, the CCPSCSS‐PF total mean scores decreased significantly, and this drop was statistically significant. When the results were analysed, it was found that as the GMFCS level elevated, the self‐care skills of children declined significantly (*p* < 0.001) (Figure 4). This suggested that the scale was valid according to the known groups' validity.

## 4 Discussion

Content and construct validity was analysed in this study, but criterion validity could not be used since there was no appropriate scale that could be applied to the sample group.

In the study, the item content validity index (I‐CVI) of the scale items ranged between 0.80–1.00, and the content validity index (S‐CVI) of the scale was 0.91. This study reported that the scale had content validity since the S‐CVI of the scale was greater than 0.80 (Esin 2018).

The KMO coefficient provides information about the fit of the data matrix for factor analysis and the fit of the data structure for factor extraction. For factor analysis, KMO is expected to exceed 0.60 (Büyüköztürk 2014). The study revealed that the KMO coefficient was 0.948 and Bartlett's test result was significant. This study result suggested that the sample size was suitable for factor analysis.

In the study, it was found that there was a construct with two factors explaining 83.4% of the total variance in CCPSCSS‐PF. The higher the explained variance, the better the related concept or construct is measured (Büyüköztürk 2014). The communality values of the two factors where the scale items are collected ranged from 0.713 to 0.908. This study result indicated that the two factors in the CCPSCSS‐PF together explained the majority of the total variance in the items and the variance related to the scale, thereby establishing construct validity.

Factor loading value is a coefficient that explains the correlation between the items and the factors. Items are expected to have high loadings on the factor they are included in (Büyüköztürk 2014). The literature indicates that items with factor loadings less than 0.40 should be removed from the scale (DeVellis and Thorpe 2021; Shrestha 2021), and according to the EFA results, 11 items (i4, i5, i9, i17, i18, i19, i20, i23, i28, i29, i30) with factor loadings less than 0.40 and no clear factor construct were removed from the analysis (Costello and Osborne 2005). Regardless of the sign, a loading value greater than or equal to 0.60 can be defined as high, and a loading value ranging from 0.30 to 0.59 can be defined as moderate (Büyüköztürk 2014). According to the EFA results of this study, the factor loadings of the two factors of the CCPSCSS‐PF were both more than 0.60 and had a high magnitude. This study result indicated that the scale satisfied the construct validity (Büyüköztürk 2014; Esin 2018).

In the literature, it is stated reports that model fit indicators should be GFI, AGFI, CFI, TLI, IFI, and IFI > 0.90, RMSEA < 0.08, and chi‐square/degree of freedom (*χ* ^2^/Df) < 5 (Esin 2018; Kline 2015; Montoya and Edwards 2021). The GFI, CFI, TLI, and IFI values in this study were greater than 0.95, the AGFI value was greater than 0.90, and *χ* ^2^/Df < 5. On the other hand, the RMSEA value was 0.097, indicated to be within acceptable limits in the literature (Browne and Cudeck 1993; Esin 2018). In general, the fit indices showed that the construct of the scale had a very good fit, and the model was compatible with the objectives of the study.

The CFA results showed that the factor loadings of the two‐factor scale were greater than or equal to 0.60 for all of the scale items except for the seventh and sixth items in Factor 2. Since the explanatory factor loading of the seventh and sixth items under Factor 2 was greater than 0.60, it was well correlated with the corrected item‐total, and the factor loading in CFA was positive and significant, which indicated that it was fully correlated with Factor 2 and the scale; thus, it was not removed from the scale. The EFA and CFA of this scale confirmed its construct validity and showed that the CCPSCSS‐PF is a valid tool that can be applied to the Turkish population.

One of the most frequently followed methods to establish the reliability of a scale is Cronbach's Alpha and Omega coefficients. These coefficients are also a measure of internal consistency. For a scale to be reliable, its reliability coefficient should be greater than or equal to 0.70 (Christmann and Van Aelst 2006; Büyüköztürk 2014). The total internal consistency Cronbach's Alpha value of the CCPSCSS‐PF and the Cronbach's Alpha values of its two subscales were greater than 0.90. These results suggest that the scale is reliable.

The reliability of the test–retest shows the consistency of the results obtained when the assessment tool is applied to the same sample group at different times. The correlation coefficient between test–retest scores should be at least 0.70 (Shrestha 2021; Esin 2018). The present study revealed that the test–retest correlation value of the scale was positive and greater than 0.70. This shows that the reliability of the CCPSCSS‐PF is high and the results are similar in both baseline and repeated assessments.

One of the methods suggested to test the validity of scales is the comparison between the known groups (Esin 2018). GMFCS levels, which assess the independence level of the child with CP in gross motor functions and are divided into five levels, for the comparison between the known groups were used in this study. While GMFCS 1 means minimal impairment for children with CP, GMFCS 5 means severe impairment. In this study, both the total scale mean score and the self‐care subscale mean score of children at GMFCS Level 1 were significantly higher than those at GMFCS Level 2, GMFCS Level 3, GMFCS Level 4, and GMFCS Level 5 for the overall CCPSCSS‐PF and Factor 1. For Factor 2, the mean score of children at GMFCS Level 5 on the subscale of the ability to express self‐care was significantly lower than the other levels. The study by Yavuz and Çimen (2007) reported that the self‐care scores of children with CP with mild spasticity were the highest and those with severe spasticity were the lowest. These results suggest that CCPSCSS‐PF can differentiate known groups.

The results of the reliability analysis in this study show that the information provided by the scale is consistent, the results are accurate, and the same results would be obtained in a second assessment with the same purpose. These results show that the scale can accurately assess the self‐care level of children with CP.

Our newly developed CCPSCSS‐PF is the only scale that assesses self‐care in children with CP aged 7–18 years and is a comprehensive and appropriate scale for use in hospitals and rehabilitation centres. To the best of our knowledge, there is no scale in the literature with proven validity and reliability that can be used to assess the self‐care skills of children with CP aged 7–18 years. The CCPSCSS‐PF can be used by professionals interested in child health, development, and education, primarily paediatric nurses, to determine the ability of children with CP aged 7–18 years to perform self‐care skills and to make interventions to improve these skills.

### 4.1 Strengths and Limitations

The limitations of the study are as follows: (1) Only volunteer parents who could be reached at special training and rehabilitation centres could participate in the study. Children who did not visit special education and rehabilitation centres and their parents could not be reached in the study. This may affect the generalisability of the scale. (2) Also, the cut‐off point of the scale could not be calculated due to the lack of a gold standard. Therefore, the self‐care levels of children with CP cannot be categorised as low or high. (3) Since children diagnosed with CP were a special group in the present study and the age range criterion was taken into account, the sample size was determined by taking ten times the number of scale items. Considering the limited literature on this subject, studies are necessary to verify the applicability of the CCPSCSS‐PF.

## 5 Conclusion

The CCPSCSS‐PF was developed to assess the self‐care skills of children with CP aged 7–18 years. The reliability and validity of the scale we developed in this study were tested. Based on these results, the CCPSCSS‐PF, which has a total of 19 items and two subscales, can (1) test the self‐care of children with CP aged 7–18 years, (2) determine the self‐care performance of children with CP aged 7–18 years who are hospitalised or who visit rehabilitation centres for education and treatment, and (3) can be used by professionals who are interested in paediatrics or working in this field, as well as undergraduate and graduate students.

It is recommended that future studies be conducted in different regions with a larger sample group to determine the self‐care skills of children with CP aged 7–18 years.

## 6 Implications for Practice

GMFCS levels, as well as other problems accompanying CP, affect the ability of children with CP to perform their self‐care skills. Children with CP may need longer than healthy children to perform their self‐care skills that require fine motor skills, such as dressing and feeding.

Parents often perform self‐care skills such as feeding, putting on and taking off clothes instead of creating appropriate opportunities for their children to perform such skills in order to manage their daily chores. Some parents may not even be aware of the existing or potential self‐care skills of their child. This scale can serve as a guiding tool for parents to determine the self‐care skills that their child with CP can perform, to recognise the self‐care skills that can be improved, and to seek professional support.

## Author Contributions

B.Y., B.K.B. and H.Ç. were responsible for data collection, data analysis, and manuscript writing. B.Y., B.K.B. and H.Ç. were responsible for subject design and manuscript review. All authors agree with the final version of the manuscript and the order in which the author names appear. This manuscript has not been published or submitted for publication in any other journal.

## Funding

The authors have nothing to report.

## Disclosure

Study statistics: The authors have checked to make sure that our submission conforms as applicable to the Journal's statistical guidelines *described here*.

*The statistics were checked prior to submission by an expert statistician, and state their name* Assoc. Prof. Dr. Emre Dünder and email: emre.dunder@omu.edu.tr.

## Ethics Statement

This study was conducted in accordance with the Helsinki Declaration and was approved by Aydın Adnan Menderes University Nursing Faculty Non‐Interventional Ethics Committee (Date: 22.05.2023, Number: 2023/352).

## Conflicts of Interest

The authors declare no conflicts of interest.

## Supporting information


**Data S1:** STROBE checklist.

## Data Availability

The data that support the findings of this study are available from the corresponding author upon reasonable request.

## References

[nop270477-bib-0001] Bobath Terapistleri Derneği [Bobath Therapists Association] . 2024. “Fonksiyonel Sınıflandırma Sistemleri [Functional Classification Systems].” Accessed December 6, 2024. https://www.bobathterapistleri.org/Pages1_2/2118.

[nop270477-bib-0002] Browne, M. W. , and R. Cudeck . 1993. “Alternative Ways of Assessing Model Fit.” In Testing Structural Equation Models, edited by K. A. Bollen and J. S. Long , 136–162. Sage.

[nop270477-bib-0025] Büyüköztürk, Ş. 2014. Sosyal bilimler için veri analizi el kitabı istatistik, araştırma deseni SPSS uygulamaları yorum [Handbook of data analysis for social sciences statistics, research design SPSS applications and interpretation]. 8th ed, 179–194. Pegem A Yayıncılık.

[nop270477-bib-0003] Chen, C. L. 2019. “Developmental Trajectory of Self‐Care in Children With Cerebral Palsy With Different Manual Abilities.” Developmental Medicine and Child Neurology 61, no. 5: 508. 10.1111/dmcn.14078. 30346035

[nop270477-bib-0004] Christmann, A. , and S. Van Aelst . 2006. “Robust Estimation of Cronbach's Alpha.” Journal of Multivariate Analysis 97, no. 7: 1660–1674.

[nop270477-bib-0005] Cohen, J. 1988. Statistical Power Analysis for the Behavioral Sciences. 2nd ed. Routledge.

[nop270477-bib-0006] Cook, J. E. , M. M. Tovin , and L. K. Kenyon . 2022. “Understanding the Lived Experience of Caring for a Child With Severe Cerebral Palsy: A Critical Step Toward Psychologically Informed Family‐Centered Care.” Physical Therapy 102, no. 4: pzab294. 10.1093/ptj/pzab294. 34972870

[nop270477-bib-0007] Costello, A. B. , and J. W. Osborne . 2005. “Best Practices in Exploratory Factor Analysis: Four Recommendations for Getting the Most From Your Analysis.” Practical Assessment, Research & Evaluation 10, no. 7: 1–9. https://scholarworks.umass.edu/pare/vol10/iss1/7.

[nop270477-bib-0008] de Leeuw, M. J. , F. C. Schasfoort , B. Spek , et al. 2021. “Factors for Changes in Self‐Care and Mobility Capabilities in Young Children With Cerebral Palsy Involved in Regular Outpatient Rehabilitation Care.” Heliyon 7, no. 12: e08537. 10.1016/j.heliyon.2021.e08537. 34950787 PMC8671866

[nop270477-bib-0009] DeVellis, R. F. , and C. T. Thorpe . 2021. Scale Development: Theory and Applications. Sage publications.

[nop270477-bib-0010] Esin, N. M. 2018. “Veri Toplama Yöntem ve Araçları &Veri Toplama Araçlarının Güvenirlik ve Gecerliği [Data Collection Methods and Tools & Reliability and Validity of Data Collection Tools].” In Hemşirelikte Araştırma Süreç, Uygulama ve Kritik [Research in Nursing: Process, Practice and Critique], edited by S. Erdoğan , N. Nahcivan , and M. N. Esin , 217–229. Nobel Tıp Kitabevleri.

[nop270477-bib-0011] Ferriero, K. , and P. Arn . 2020. “Genetic Abnormalities and Congenital Malformations as a Cause of Cerebral Palsy.” In Cerebral Palsy, edited by F. Miller , S. Bachrach , N. Lennon , and M. E. O'Neil . Springer. 10.1007/978-3-319-74558-9_2.

[nop270477-bib-0012] Fritz, H. , and C. Sewell‐Roberts . 2020. “Family Stress Associated With Cerebral Palsy.” In Cerebral Palsy, edited by F. Miller , S. Bachrach , N. Lennon , and M. E. O'Neil . Springer. 10.1007/978-3-319-74558-9_213.

[nop270477-bib-0013] Himmelmann, K. , and C. P. Panteliadis . 2018. “Clinical Characteristics.” In Cerebral Palsy, edited by C. Panteliadis . Springer. 10.1007/978-3-319-67858-0_10.

[nop270477-bib-0014] Holgado‐Tello, F. P. , S. Chacón–Moscoso , I. Barbero–García , and E. Vila–Abad . 2010. “Polychoric Versus Pearson Correlations in Exploratory and Confirmatory Factor Analysis of Ordinal Variables.” Quality & Quantity 44: 153–166. 10.1007/s11135-008-9190-y.

[nop270477-bib-0015] Kahraman Berberoğlu, B. , and H. Çalışır . 2020. “Serebral Palsili Bir Çocuğun Orem Öz‐Bakım Eksikliği Kuramı'na Göre Hemşirelik Bakımı: Olgu Sunumu [Nursing Care of A Child With Cerebral Palsy According to the Orem Self‐Care Deficiency Theory: Case Report].” Adnan Menderes Üniversitesi Sağlık Bilimleri Fakültesi Dergisi 4, no. 2: 154–167. 10.46237/amusbfd.613380.

[nop270477-bib-0016] Kline, R. B. 2015. Principles and Practice of Structural Equation Modeling. 2nd ed. Guilford Press.

[nop270477-bib-0017] Kunz Stansbury, J. C. , and D. Wilson . 2015. “The Child With Neuromuscular or Muscular Dysfunction.” In Wong's Essentials of Pediatric Nursing, edited by M. J. Hockenberry and D. Wilson , 10th ed., 1617–1628. Mosby&Elseiver.

[nop270477-bib-0018] Montoya, A. K. , and M. C. Edwards . 2021. “The Poor Fit of Model Fit for Selecting Number of Factors in Exploratory Factor Analysis for Scale Evaluation.” Educational and Psychological Measurement 81, no. 3: 413–440. 10.1177/0013164420942899. 33994558 PMC8072951

[nop270477-bib-0019] Patil, I. 2021. “ggstatsplot: ‘ggplot2’ Based Plots with Statistical Details.” https://CRAN.R‐project.org/package=ggstatsplot.

[nop270477-bib-0020] Sadowska, M. , B. Sarecka‐Hujar , and I. Kopyta . 2020. “Cerebral Palsy: Current Opinions on Definition, Epidemiology, Risk Factors, Classification and Treatment Options.” Neuropsychiatric Disease and Treatment 16: 1505–1518. 10.2147/NDT.S235165. 32606703 PMC7297454

[nop270477-bib-0021] Shrestha, N. 2021. “Factor Analysis as a Tool for Survey Analysis.” American Journal of Applied Mathematics and Statistics 9, no. 1: 4–11. 10.12691/ajams-9-1-2.

[nop270477-bib-0022] Yavuz, B. 2006. “Investigation of the Fulfilment Levels of the Self‐Care Skills of the Children With Cerebral Palsy and Affectional Factors.” Post Graduate thesis, Dokuz Eylül University, İzmir.

[nop270477-bib-0023] Yavuz, B. , and S. Çimen . 2007. “Serebral Palsili Çocukların öz Bakım Becerilerini Gerçekleştirme Düzeyleri ve Etkileyen Etmenlerin Incelenmesi [Investigation of the Fulfilment Levels of the Self‐Care Skills of the Children With Cerebral Palsy andAffectional Factors].” Cumhuriyet Üniversitesi Hemsirelik Yüksekokulu Dergisi 11, no. 1: 17–25.

[nop270477-bib-0024] Zahavi, D. , and K. M. Martiny . 2019. “Phenomenology in Nursing Studies: New Perspectives.” International Journal of Nursing Studies 93: 155–162. 10.1016/j.ijnurstu.2019.01.014. 30795899

