# Correction to ‘Psychometric Properties of the Children With Cerebral Palsy (7–18 Years Old) Self‐Care Skills Scale‐Parent Form: A Turkish Validity and Reliability Study’

**DOI:** 10.1002/nop2.70786

**Published:** 2026-09-15

Yavuz, Betul , Bircan Kahraman Berberoğlu , and Hüsniye Çalişir . 2026. “Psychometric Properties of the Children With Cerebral Palsy (7–18 Years Old) Self‐Care Skills Scale‐Parent Form: A Turkish Validity and Reliability Study.” Nursing Open 13, no. (3): e70477. 10.1002/nop2.70477.41857787 PMC13097517

This paper was originally published as a “Research Methodology: Discussion Paper ‐ Methodology” article type, but has now been corrected to an “Empirical Research Quantitative”.

We apologize for this error.

